# Left adrenal aldosteronism coexisting with left paraaortic paraganglioma presenting as bilateral adrenal and left paraaortic tumors– comprehensive adrenal evaluation aiding perfect management: a case report

**DOI:** 10.1186/s12902-022-01181-6

**Published:** 2022-11-12

**Authors:** Yu-Chen Hsu, Cheng-Han Lee, Chen-Yu Chen, Chung-Jye Hung

**Affiliations:** 1 Department of Obstetrics and Gynecology, College of Medicine, National Cheng Kung University Hospital, National Cheng Kung University, No. 138, Sheng-Li Road, Tainan, Taiwan; 2 Department of Surgery, College of Medicine, National Cheng Kung University Hospital, National Cheng Kung University, No. 138, Sheng-Li Road, Tainan, Taiwan

**Keywords:** Paraganglioma, Hyperaldosteronism, Metaiodobenzylguanidine scan, Adrenal venous sampling, Case report

## Abstract

**Background:**

Coexistence of a catecholamine-secreting tumor and an adrenal cortical tumor is quite rare which makes both diagnosis and management challenging. The purpose of this article is to describe the presence of this condition, share a stepwise approach for preoperative evaluation, and review the related literature.

**Case presentation:**

A 44-year-old male patient had a history of hypertension and aggravating hypokalemia for years. Abdominal computed tomography incidentally found concomitant bilateral adrenal and left para-aortic tumors. Comprehensive adrenal hormone tests revealed a high aldosterone renin ratio and mildly elevated 24-h urine vanillylmandelic acid and norepinephrine levels. Subsequently, a metaiodobenzylguanidine scan showed uptake over the left para-aortic tumor, and NP-59 adrenal scintigraphy showed uptake over the left adrenal tumor. Further confirmatory tests, including captopril suppression, irbesartan suppression, and saline infusion, all confirmed the diagnosis of hyperaldosteronism. Adrenal venous sampling following 2 months of preparation with an alpha blocker demonstrated a left aldosterone-producing adrenal adenoma. Combining hormonal analysis, imaging studies, and adrenal venous sampling, the patient was diagnosed with left adrenal aldosteronoma, right adrenal nonfunctional tumor, and left para-aortic paraganglioma (PGL). Accordingly, laparoscopic left adrenalectomy and left PGL excision were performed smoothly under alpha blocker maintenance. The pathology report confirmed left adrenal cortical adenoma and left para-aortic PGL. Postoperatively, the blood pressure, biochemical tests, and adrenal hormone assays returned to normal, and related symptoms disappeared and were relatively stable during the follow-up period of two years.

**Conclusions:**

This is the first case of left para-aortic PGL coexisting with an ipsilateral aldosterone-producing adenoma presenting as a left para-aortic tumor associated with bilateral adrenal tumors. Awareness of the rarity of this coexistence can avoid unexpected disasters during the process of evaluation and management.

## Background

Among all hypertensive patients, primary hyperaldosteronism is frequently diagnosed with a prevalence varying from 3.2% to 12.7% in primary care [1]. Unilateral aldosterone-producing adenoma accounts for approximately 30% of all primary hyperaldosteronism [2]. Although the incidence of primary aldosteronism among bilateral adrenal incidentaloma is low [3, 4], the incidence of unilateral aldosterone-producing adenoma presenting as bilateral adrenal tumors is approximately 20% [5]. In addition, the treatment of choice for primary aldosteronism with bilateral adrenal tumors depends on the distinction between bilateral adrenal hyperplasia and unilateral aldosterone-producing adenoma [6]. According to the Endocrine Society Guidelines, patients with bilateral adrenal hyperplasia should be treated with medical treatments, while the recommendation for patients with unilateral hyperaldosteronism is to undergo surgical resection [7]. Therefore, the lateralization of bilateral adrenal tumors is undoubtedly important. As per expert consensus guidelines, adrenal venous sampling remains a standard tool for identification of surgically curable causes of primary aldosteronism, although it is technically challenging [8].

The incidence of pheochromocytoma and paraganglioma (PGL) has been reported to be approximately 0.6 cases per 100,000 person-years in the States [9] and 0.04 to 0.21 cases per 100,000 person-years in the Netherlands [10]. Among all catecholamine-secreting tumors, 80–85% are pheochromocytomas, and 15–20% are PGLs [11]. In addition to the rarity of pheochromocytomas and PGLs, they have been described as the “great mimic” for their variety of clinical symptoms [12]. The classical triad of symptoms includes headache, palpitations, and sweating, yet these are present in less than 25% of patients [11]. Hypertension is found in 80–90% of patients and half of those have paroxysmal hypertension [13]. Approximately 35% of pheochromocytoma or PGL cases are of inherited origin [14]. Catecholamine-secreting tumors remain a frequently overlooked diagnosis [15], and nearly 50% of deaths in patients with unsuspected tumors occur during anesthesia, surgery, or parturition [16]. The factors triggering an intraoperative crisis can be attributed to excessive release of catecholamines from the undiagnosed tumor secondary to the patient’s anxiety, induction or intubation of the anesthesia, and direct or indirect manipulation of the tumor [16]. Therefore, detecting and diagnosing catecholamine-secreting tumors before any precipitating events occur is of utmost importance.

The coexistence of catecholamine-secreting tumors and adrenal cortical tumors is quite rare and leads to not only diagnosis but also management challenges. Here, a case of para-aortic PGL coexisting with an ipsilateral aldosterone-producing adenoma presenting as left para-aortic tumor associated with bilateral adrenal tumors, following a stepwise evaluation using up-to-date adrenal diagnostic modalities that leads to a successful surgical intervention, is reported, and the related literature is reviewed.

## Case presentation

**Fig. 1:**
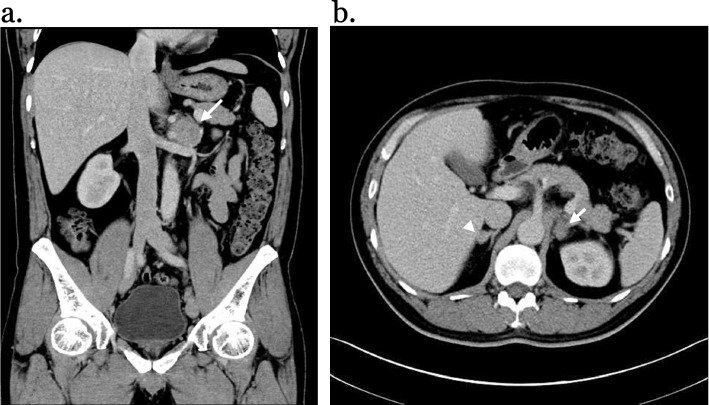
Abdominal computed tomography showed (**a**) one 4.3 cm left para-aortic tumor (arrow) surrounded by the aorta, celiac trunk, superior mesenteric artery, and renal vessels, with the Hounsfield unit being approximately 40 under the noncontrast phase and 70 under the arterial phase and (**b**) a 2.1 cm left adrenal tumor (arrow) and a 1.2 cm right adrenal tumor (arrowhead)

A 44-year-old male patient presented with years of hypertension (since 2012) and received regular follow-up and medical treatment at a cardiovascular outpatient clinic. Some nonspecific symptoms, including chest tightness, palpitations, flushing, and bilateral leg weakness, were described. Neither headache nor sweating was complained. According to the guidelines from the National Committee on Prevention, Detection, Evaluation, and Treatment of High Blood Pressure [17], indications for evaluation of secondary hypertension include resistant hypertension, age of onset before puberty, age younger than 30 years with nonobesity and no family history, malignant hypertension, or acute rise of blood pressure in previous stable readings. Therefore, a more detailed assessment of the possible underlying cause of the hypertension was not done between 2012–2016. It was not until 2016 that hypokalemia was first noted. From 2016 to 2019, there were episodes of recurrent hypokalemia despite potassium supplementation. The potassium level was approximately 3.1 mmol/L (normal, 3.5–5.1 mmol/L) with a high potassium diet during 2016–2018 but decreased to 2.7 mmol/L in 2018. Hypokalemia-associated drugs, such as thiazide diuretics, were then discontinued. However, the potassium level dropped to 2.3 mmol/L in 2019, and intravenous potassium supplementation was given twice. To investigate the cause of hypokalemia, several blood and urinary examinations were performed. A trans-tubular potassium gradient of 10.5 (> 7) and urine potassium/creatinine ratio of 68 (> 1.5) indicated renal loss of potassium. Venous gas analysis reported metabolic alkalosis, which ruled out renal tubular acidosis or other causes of metabolic acidosis. Given renal potassium loss accompanied by refractory hypertension, hyperaldosteronism was therefore suspected. Elevated plasma aldosterone levels of 489 pg/ml (normal, 68.0–173.0 pg/ml), low plasma renin concentrations of 2.89 pg/ml (normal, 1.1–20.2 pg/ml), and elevated aldosterone renin ratios (ARRs) of 169.2 were strongly indicated primary aldosteronism. Further abdominal computed tomography incidentally found bilateral adrenal tumors (right: 1.2 cm, left: 2.1 cm) and a left para-aortic tumor (4.3 cm), with the Hounsfield unit of the para-aortic tumor being approximately 40 under the noncontrast phase and 70 under the arterial phase, which suggested many possibilities (Fig. 1). Upon referral to the surgical outpatient clinic, his blood pressure was approximately 155/105 mmHg with receiving three types of antihypertensives, including calcium channel blocker/angiotensin receptor blocker (amlodipine/valsartan (5/80 mg) 1# bid, nifedipine (10 mg) 1# prn) and an alpha blocker (doxazosin (4 mg) 1# qd). Reviewing his family history, no endocrine disorders were identified. To clarify the nature of the bilateral adrenal tumors, comprehensive adrenal hormone tests were performed, including serum cortisol, adrenocorticotropic hormone (ACTH), 1 mg overnight dexamethasone suppression test (ODST), dehydroepiandrosterone (DHEA), and 24-h urine vanillylmandelic acid (VMA) and catecholamines. The results showed normal cortisol levels of 12.6 µg/dl (normal, 2.9–17.3 µg/dl), ACTH levels of 34.5 pg/ml (normal, 7.2–63.3 pg/ml), DHEA levels of 1464 ng/ml (normal, 215–2769 ng/ml), suppressive ODST tests of cortisol levels < 1 µg/dl (normal, < 1.8 μg/dl), mildly elevated 24-h urine VMA levels of 8.61 mg/day (normal, 1–7.5 mg/day), norepinephrine levels of 105.0 µg/day (normal, 12.1–85.5 µg/day), and normal epinephrine levels of 16.5 µg/day (normal, 0–22.4 µg/day). Metanephrine and normetanephrine were not available in our lab. Mild elevation of 24-h urine VMA and catecholamines suggested more possibilities for both adrenal and para-aortic lesions that could be para-aortic PGL, unilateral or bilateral pheochromocytoma (although less likely), or neither.

**Fig. 2:**
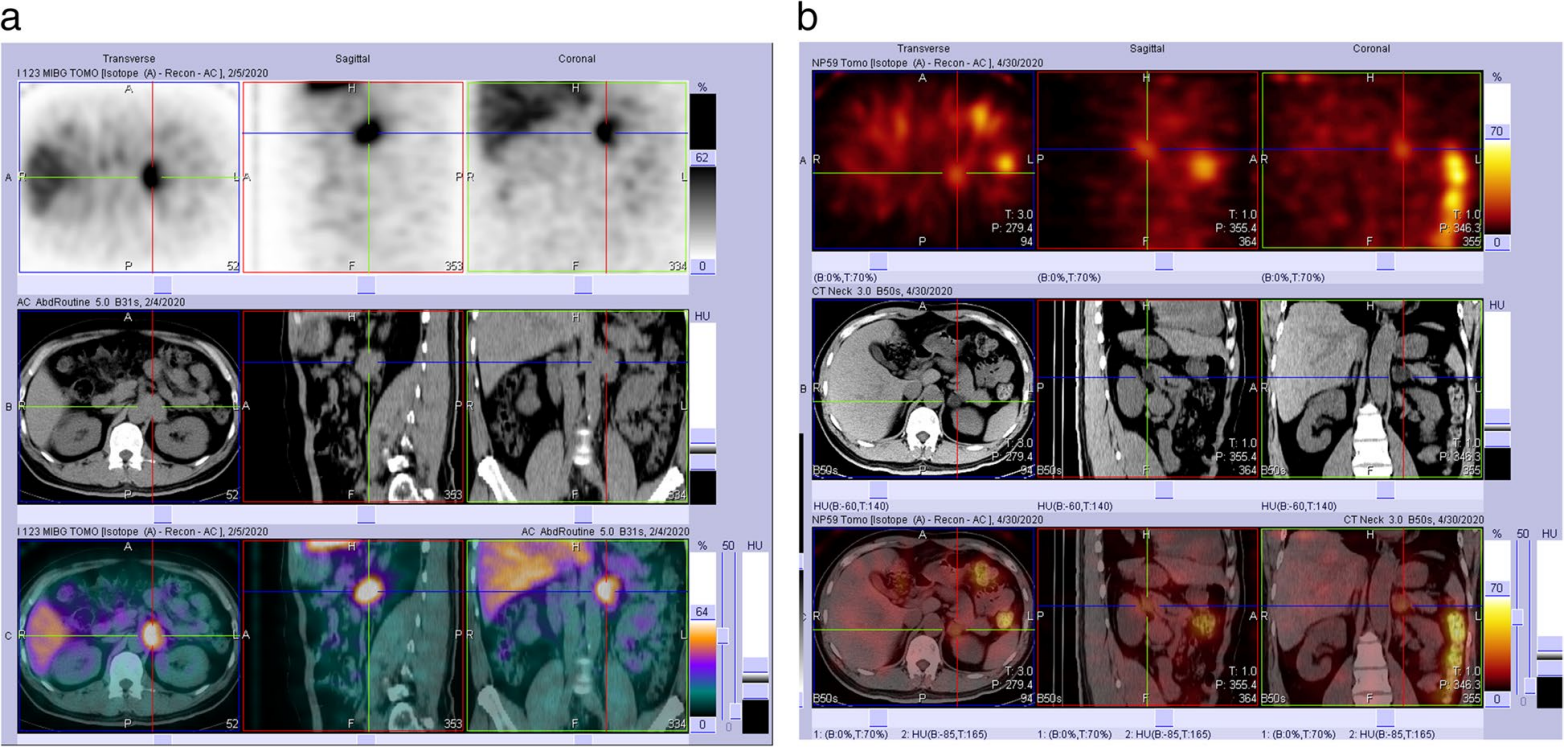
Metaiodobenzylguanidine scan (**a**) showed uptake over the left para-aortic tumor, which favored paraganglioma, and NP-59 adrenal scintigraphy (**b**) showed mild radiotracer accumulation over the left supra-renal region, which favored left adrenal functional cortical tumor

To verify the hormonal function results, a MIBG scan was performed that revealed high uptake over the left para-aortic but not the bilateral adrenal tumors, indicating the diagnosis of left para-aortic PGL (Fig. 2). For suspicion of primary hyperaldosteronism, confirmatory tests, including captopril suppression, irbesartan suppression, and saline infusion were performed, and all confirmed the diagnosis. Briefly, the captopril suppression test showed a non-suppressed aldosterone level (645 pg/ml) and ARR (507) after giving 50 mg captopril for 1.5 h (a positive test is defined as aldosterone level > 100 pg/ml and ARR > 35). The irbesartan suppression test showed a non-suppressed aldosterone level (590 pg/ml) and ARR (479) after giving 75 mg irbesartan for 2 h (a positive test is defined as aldosterone level > 100 pg/ml and ARR > 35). The saline infusion test showed a non-suppressed aldosterone level (540 pg/ml) after 2 L of intravenous saline infusion for 4 h (a positive test is defined as an aldosterone level > 100 pg/ml) (Table 1). The posture stimulation test showed a mild reduction in serum aldosterone (337 pg/ml to 302 pg/ml) after 4 h of ambulation. Subsequently, NP-59 adrenal scintigraphy revealed radiotracer accumulation over the left suprarenal region, which proved left adrenal functioning cortical adenoma (Fig. 2). For definite lateralization, adrenal venous sampling (AVS) was performed after 2 months of alpha blocker preparation (doxazosin (4 mg) shifted to terazosin (2 mg), terazosin dose titration from 0.5# to 3# hs) accompanied with calcium channel blocker/angiotensin receptor blocker (amlodipine/valsartan (5/80 mg) 1# bid, nifedipine (10 mg) 1# prn). The blood pressure before AVS was approximately 140/95 mmHg and jumped to 170/110 mmHg during the procedure in the short term. Left aldosterone-producing adrenal adenoma was confirmed by AVS data analysis (Table 2). A potassium-sparing diuretic (spironolactone (25 mg) 1# bid) was added following AVS with maintenance of the calcium channel blocker/angiotensin receptor blocker (amlodipine/valsartan (5/80 mg) 1# bid) and an alpha blocker (terazosin (2 mg) 3# hs). Combining hormonal analysis, imaging studies, and adrenal venous sampling, the patient was diagnosed with left adrenal aldosteronoma, right adrenal nonfunctional tumor, and left para-aortic PGL.

**Table 1:** Confirmatory test for hyperaldosteronism

	**Cortisol (µg/dl)**	**K (mmol/L)**	**PRC (pg/ml)**	**Aldosterone (pg/ml)**
Captopril suppression test (50 mg)				
Before			1.58	654
1 h later			2.10	749
1.5 h later			1.27	645
Irbesartan suppression test (75 mg)				
Before			2.60	810
2 h later			1.23	590
Saline infusion test (2 L)				
Before	10.9	2.5	1.81	627
4 h later	7.86	2.9	< 1.0	540

*PRC* Plasma renin concentration

**Table 2:** Adrenal venous sampling result

AVS^a^	IVC	Right	Left
**Pre-ACTH stimulation**			
Aldosterone (pg/ml)	1036/1080	676/625	9219/9319
Cortisol (μg/dl)	13.80/13.70	19.10/18.90	47.36/43
Aldosterone/Cortisol	75.07/78.83	35.39/33.07	194.90/216.72
Selectivity index		1.38^b^/1.38^b^	3.43/3.14
Relative secretion index		0.47/0.42	2.60/2.75
Lateralization index			5.51/6.55
Contralateral suppression index		0.47/0.42	
**Post-ACTH stimulation**			
Aldosterone (pg/ml)	1418/1222	6252/5719	12,388/19962
Cortisol (μg/dl)	17.90/18.30	937/968	346/506
Aldosterone/Cortisol	79.22/66.78	6.67/5.91	35.8/39.45
Selectivity index		52.35/52.90	19.32/27.65
Relative secretion index		0.08/0.09	0.45/0.59
Lateralization index			5.63/6.56
Contralateral suppression index		0.08/0.09	

*AVS* Adrenal venous sampling, *IVC* Inferior vena cava, *ACTH* Adrenal corticotropic hormone, *Selectivity index* $\left(\frac{{\mathrm{Cortisol}}_{\;\mathrm{side}}}{{\mathrm{Cortisol}}_{\;\mathrm{IVC}}}\right)$, *Relative secretion index* $\left(\frac{{\mathrm{Aldosterone}}_{\;\mathrm{side}}/{\mathrm{Cortisol}}_{\;\mathrm{side}}}{{\mathrm{Aldosterone}}_{\;\mathrm{IVC}}/{\mathrm{Cortisol}}_{\;\mathrm{IVC}}}\right)$, *Lateralization index* $\left(\frac{\mathrm{Aldosterone}\;_{\mathrm{dominant}}/{\mathrm{Cortisol}}_{\;\mathrm{dominant}}}{{\mathrm{Aldosterone}}_{\;\mathrm{nondominant}}/{\mathrm{Cortisol}}_{\;\mathrm{nondominant}}}\right)$, *Contralateral suppression index* $\left(\frac{{\mathrm{Aldosterone}}_{\;\mathrm{nondominant}}/{\mathrm{Cortisol}}_{\;\mathrm{nondominant}}}{{\mathrm{Aldosterone}}_{\;\mathrm{IVC}}/{\mathrm{Cortisol}}_{\;\mathrm{IVC}}}\right)$

^a^Adrenal venous sampling was done twice before and after ACTH stimulation and revealed left lateralization by lateralization index and contralateral suppression index

^b^Successful catheterization confirmed except pre-ACTH stimulation right adrenal vein sampling (positive cutoff value > 2x)

**Fig. 3:**
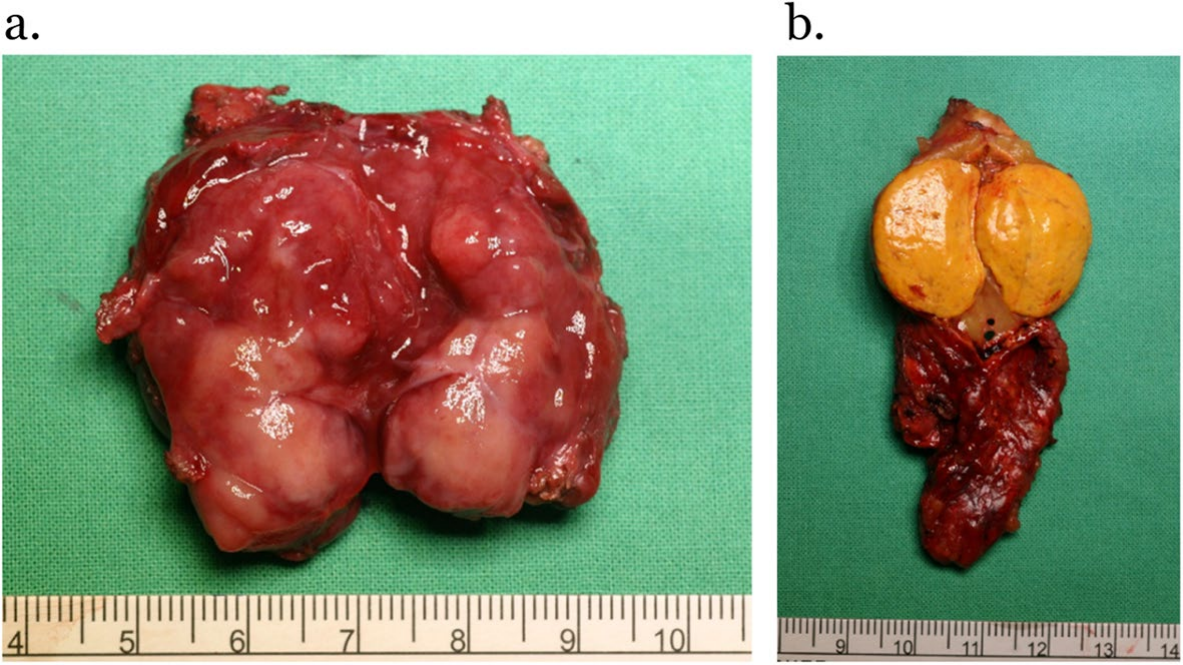
Operative findings showed (**a**) a 4.5*4.5*2.2 cm soft left para-aortic flesh tumor and (**b**) a 2.4*2.0*2.0 cm soft homogeneous yellowish left adrenal cortical tumor

Accordingly, laparoscopic left adrenalectomy followed by para-aortic PGL excision were performed under the use of antihypertensives (spironolactone (25 mg) 1# bid, amlodipine/valsartan (5/80 mg) 1# bid, and terazosin (2 mg) 3# hs) with a preoperative blood pressure of approximately 130/90 mmHg. Operative findings showed one 2.4*2.0*2.0 cm soft homogenous yellowish left adrenal cortical tumor and one 4.5*4.5*2.2 cm para-aortic flesh tumor surrounded by renal vessels, the aorta, celiac trunk, and superior mesenteric artery (Fig. 3). Hyperglycemia (> 500 mg/dl), metabolic acidosis, and systolic blood pressure elevation to 160 mmHg occurred during manipulation of the para-aortic PGL, and systolic blood pressure dropped to approximately 80 mmHg after removal of the para-aortic PGL. The patient was sent to the intensive care unit for postoperative care. Hyperglycemia and metabolic acidosis were controlled with insulin and intravenous fluid supplementation. Extubation was successful on the 2^nd^ postoperative day, and hypokalemia was corrected in two days. The patient was then transferred to the general ward with a stable condition on the third postoperative day.

The final pathology revealed adrenal cortical adenoma and para-aortic PGL with rare mitotic figures and absent necrosis. Furthermore, endocrine function evaluations were performed for the potential related components of multiple endocrine neoplasia, including calcitonin, carcinoembryonic antigen, calcium, phosphorus, parathyroid hormone, growth hormone, and prolactin, which were all normal. Sonography reported no identified thyroid or parathyroid lesions. Genetic testing, including ARNT (HIF1β), ATRX, BRAF, CSDE1, EGLN1 (PHD2), EPAS1 (HIF1α), FGFR1, HRAS, IDH1, MAML3, MAX, NF1, NGFR, RET, SDHA, SDHAF2, SDHB, SDHC, SDHD, SETD2, TMEM127, TP53, VHL, CDKN2A, H3F3A, IDH2, KMT2D, MDH2C, MERTK, and MET, using next-generation sequencing revealed no major genetic variants, although gene variants related to large genome insertion, large genome deletion, inversion, and complicated rearrangement may not be totally excluded.

His blood pressure returned to approximately 120/85 mmHg with all the antihypertensive drugs being withdrawn three months after the operation. Urine VMA, catecholamine profile, and serum aldosterone, renin, and potassium levels returned to normal quickly postoperatively and have remained relatively stable. The patient had no symptoms of bilateral leg weakness, chest tightness, or palpitations during the follow-up period of two years.

### Discussion and conclusions

The coexistence of catecholamine-secreting tumors and adrenal cortical tumors is extremely rare and the manifestation of this combination requires further description. Regarding this combination, catecholamine-secreting tumors can be either pheochromocytomas or PGLs, and adrenal cortical tumors can be aldosterone-secreting, Cushing or subclinical Cushing tumors, hyperandrogenic, or nonfunctioning tumors. Regarding manifestation, the coexistence of catecholamine-secreting tumors and adrenal cortical tumors can manifest as ipsilateral or contralateral tumors, and each tumor can manifest as unilateral or bilateral. From the literature reported after 1985, there were 38 cases of pheochromocytoma coexisting with adrenal cortical tumors, including 13 cases of hyperaldosteronism [18–25], 8 cases of Cushing’s or subclinical Cushing’s tumors [14, 18, 26–28], 1 case of hyperandrogenism [29], 6 cases of nonfunctioning adrenal tumors [30–34], and 10 cases of unknown functional entities [18, 35]. Furthermore, there have been only 4 cases of PGL coexisting with unilateral adrenal cortical tumor, including 3 cases of hyperaldosteronism [23, 36, 37] and one case of adrenal cortical carcinoma [38]. The case presented is of a left aldosterone-producing adenoma coexisting with ipsilateral para-aortic PGL manifesting as bilateral adrenal tumors associated with a left para-aortic tumor. To our knowledge, this is the first case of this manifestation of combination following a thorough literature review. Some hypotheses have been proposed to explain the coexistence of catecholamine-secreting tumors and adrenal cortical tumors [14, 36, 39]; however, no consistent association was found by earlier research [40]. Therefore, most of the cases are still thought to be of coincidental coexistence.

The key point regarding the appropriate management of this complicated case is the challenging stepwise diagnostic approach, including the confirmation of the suspicious PGL that can mimic many presentations and the distinction between bilateral adrenal hyperplasia and unilateral aldosterone-producing adenoma– both being difficult to diagnose. Following initial hormonal screening studies, primary hyperaldosteronism was highly suspected; however, the evidence for the possible existence of a catecholamine-secreting tumor was not strong enough due to mild elevation of VMA and norepinephrine (lower than the twofold reference range). Based on the Mayo Clinic, the diagnostic cutoff point for pheochromocytoma is approximately twofold higher than the normal population reference range. Nevertheless, individuals with only mild or borderline elevations of urinary total metanephrines or catecholamines are not inappropriately labeled as potentially having a pheochromocytoma [41]. The study from Yu et al. supported the use of a 2- to threefold upper limit of normal as a value above which pheochromocytoma is likely; however, mildly elevated or normal results were 20% or 5% in the correctly diagnosed group and 17% or 17% in the underdiagnosed group, respectively [42]. The imaging phenotype consistent with pheochromocytoma includes enhancement with contrast medium on CT, high signal intensity on T2-weighted MRI, cystic and hemorrhagic changes, variable sizes, and the possibility of bilateral tumors [43–46]. The most common site of PGL is over the organ of Zuckerandl, between the inferior mesenteric artery and aortic bifurcation, the infradiaphragmatic paraaortic region, and the mediastinum [47]. From the imaging phenotype of the paraaortic tumor in our case, catecholamine-secreting tumors were also taken into consideration despite having many differential diagnoses, including metastatic tumors, lymphadenopathy, or other mesenchymal tumors. Under these considerations, although the coexistence of PGL and adrenal cortical adenoma is extremely rare, an MIBG scan was prompted and excitingly became the determining factor in examination of this patient.

Second, the potential risk during the evaluation process for primary aldosteronism in the presence of a catecholamine-secreting tumor, in contrast to its absence, needs to be addressed in this particular setting. PGL is a catecholamine-secreting tumor that can result in cardiac arrest, respiratory failure, or other associated diseases if treated incautiously [42]. Therefore, there were several treatment suggestions regarding preoperative preparation before PGL removal, and the setting of blood pressure and heart rate before the operation was also being researched [7, 48]. Noxious stimuli, such as venous catheterization, tracheal intubation, skin incision, anesthetic drugs, and palpation of the tumor or abdominal exploration, can induce a hypertensive crisis by releasing catecholamines from the tumors [49]. Adrenal venous sampling is a vital procedure, although invasive, for the differential diagnosis of unilateral and bilateral hyperaldosteronism. The issue of the necessity of alpha blocker preparation before adrenal venous sampling should be taken into serious consideration in the existence of catecholamine-secreting tumors. However, these are still extremely rare suggestions about alpha blocker preparation for invasive procedures other than surgery on patients with catecholamine-secreting tumors, not to mention adrenal venous sampling. A newly published report recommended that diagnosis of concomitant pheochromocytoma and primary aldosteronism warrants adrenal venous sampling to determine the laterality of primary aldosteronism but also failed to address the necessity of alpha blocker preparation [25]. Hasassri et al. reported that two patients with primary aldosteronism developed a hypertensive crisis during adrenal venous sampling because of lack of awareness of the coexistence of a pheochromocytoma. They suggested that in patients with mixed biochemical and/or imaging findings, adrenal venous sampling should only be conducted after a pheochromocytoma has been excluded by biochemical workup to avoid a hypertensive crisis during the procedure [18]. This report clarifies and confirms our doubt about the necessity of preparation before adrenal venous sampling in this rare setting. In our case, an alpha blocker was added, accompanied with calcium channel blocker/angiotensin receptor blocker, once the MIBG report came out, and used for approximately two months before adrenal venous sampling was done. As expected, adrenal venous sampling was performed smoothly with successful catheterization.

Lastly, there are many debates regarding the necessity and number of positive confirmatory tests needed for primary aldosteronism evaluation. Various confirmatory tests may differ in terms of sensitivity, specificity, and reliability, and the choice of confirmatory test is commonly determined by considering cost, patient compliance, laboratory routine, and local expertise [7]. The European Endocrine Society guidelines recommend that patients with a positive ARR undergo one or more confirmatory tests to definitively confirm or exclude the diagnosis and also suggest that there may be no need for further confirmatory testing in the setting of spontaneous hypokalemia, plasma renin below detection levels plus plasma aldosterone levels higher than 20 ng/dl (200 pg/ml) [7]. Therefore, in our scenario, 1 confirmatory test should be enough in daily practice and the reason to do more confirmatory tests that is available in our lab is for academic research interest.

In summary, we present a case of left para-aortic PGL coexisting with an ipsilateral aldosterone-producing adenoma presenting as a left para-aortic tumor associated with bilateral adrenal tumors. The rare combination and unprecedented presentation make the differential diagnosis complicated and challenging, including both the confirmation of the great mimic PGL and the distinction between unilateral and bilateral hyperaldosteronism. The preparation with the alpha blocker before adrenal venous sampling and operation afterward in this setting is also worth of emphasis.

## Data Availability

Clinical data from the corresponding author will be available upon request.

## Abbreviations

ACTH: Adrenocorticotropic hormone
ARR: Aldosterone renin ratio
MIBG: Metaiodobenzylguanidine
ODST: Overnight dexamethasone suppression test
PGL: Paraganglioma
VMA: Vanillylmandelic acid
